# Utilizing a Two-Stage Taguchi Method and Artificial Neural Network for the Precise Forecasting of Cardiovascular Disease Risk

**DOI:** 10.3390/bioengineering10111286

**Published:** 2023-11-04

**Authors:** Chia-Ming Lin, Yu-Shiang Lin

**Affiliations:** Professional Master Program in Artificial Intelligence in Medicine, College of Medicine, Taipei Medical University, Taipei 11031, Taiwan; g167111013@tmu.edu.tw

**Keywords:** cardiovascular disease, two-stage Taguchi optimization method, artificial neural network, point-of-care testing

## Abstract

The complexity of cardiovascular disease onset emphasizes the vital role of early detection in prevention. This study aims to enhance disease prediction accuracy using personal devices, aligning with point-of-care testing (POCT) objectives. This study introduces a two-stage Taguchi optimization (TSTO) method to boost predictive accuracy in an artificial neural network (ANN) model while minimizing computational costs. In the first stage, optimal hyperparameter levels and trends were identified. The second stage determined the best settings for the ANN model’s hyperparameters. In this study, we applied the proposed TSTO method with a personal computer to the Kaggle Cardiovascular Disease dataset. Subsequently, we identified the best setting for the hyperparameters of the ANN model, setting the hidden layer to 4, activation function to tanh, optimizer to SGD, learning rate to 0.25, momentum rate to 0.85, and hidden nodes to 10. This setting led to a state-of-the-art accuracy of 74.14% in predicting the risk of cardiovascular disease. Moreover, the proposed TSTO method significantly reduced the number of experiments by a factor of 40.5 compared to the traditional grid search method. The TSTO method accurately predicts cardiovascular risk and conserves computational resources. It is adaptable for low-power devices, aiding the goal of POCT.

## 1. Introduction

Cardiovascular diseases (CVDs) are some of the leading causes of death globally, imposing significant health and economic burdens on individuals and societies. Common CVDs include coronary artery disease, myocardial infarction, arrhythmias, heart failure (HF), stroke, and atherosclerosis. These diseases affect the normal functioning of the heart and blood vessels, leading to inadequate blood and oxygen supplies and causing severe disruptions to systems throughout the entire body. Among them, coronary artery disease is characterized by a narrowing or blockage of the coronary arteries, which are responsible for supplying oxygen and nutrients to the heart. CVDs also include arrhythmias, where the heart rhythm may become too fast, too slow, or irregular. HF refers to the inability of the heart to effectively pump blood, leading to insufficient blood and oxygen supplies to various organs, leading to symptoms such as fatigue, shortness of breath, and swelling. Stroke is another CVD that occurs when the blood flow to a specific region of the brain is interrupted by a clot or bleeding in cerebral blood vessels. This can result in impaired brain functions, such as language and motor skills. Atherosclerosis is a progressive process involving the accumulation of plaque and the hardening of arterial walls. Over time, plaques can increase, further narrowing or blocking blood vessels, restricting blood flow, and increasing the risk of CVDs [1].

CVD factors are typically interconnected. Multiple risk factors such as hypertension, hyperlipidemia, and diabetes can coexist in an individual and influence each other [2]. This comprehensive impact complicates the mechanisms underlying CVDs. Therefore, the prevention and management of CVDs require a comprehensive consideration of these risk factors and the development of corresponding prevention strategies. Additionally, CVD risk factors are closely related to lifestyle. Unhealthy lifestyle habits such as an unhealthy diet, a lack of exercise, smoking, and excessive alcohol consumption increase the risk of developing CVDs [3,4,5]. This highlights the critical role of individual choices and behaviors in cardiovascular health. Changing unhealthy lifestyle habits, following healthy dietary guidelines, increasing physical activity, quitting smoking, and limiting alcohol intake can effectively reduce the risk of developing CVDs. Early detection and management are also crucial for preventing CVDs.

Early disease detection is a challenging task. Many scholars have conducted extensive research on the early detection of various diseases [6,7,8]. However, in the early stages, Cardiovascular Diseases (CVD) typically do not display obvious symptoms. Many individuals may experience mild discomfort, such as slight fatigue or chest tightness, in the early development of the disease, and such symptoms are easily overlooked or attributed to other causes. Due to the lack of clear warning signs, people often do not proactively seek medical help, making the early detection of CVD even more difficult. Additionally, the progression of certain CVDs is covert and gradual. For example, in the early stages, atherosclerosis might not cause obvious symptoms, but, over time, fat and plaque can gradually accumulate within the blood vessels, ultimately leading to vessel blockage or myocardial ischemia. As a result, many individuals only become aware of the problem when the disease has progressed to a more severe stage, which increases the difficulty of treatment and recovery. The risk of CVDs is often influenced by interactions between multiple factors. However, individually assessing each risk factor might not comprehensively evaluate an individual’s overall risk, making it more complex to identify early signs of CVD. The early detection of CVDs requires effective screening tools and resources. Many cardiovascular examinations, such as electrocardiograms, blood tests, and cardiac ultrasounds, require specialized equipment and trained professionals for interpretation. However, in certain regions or under resource-limited conditions, these examinations might not be widely available, further increasing the difficulty of detecting CVDs early.

The effective prediction of CVDs can help physicians identify high-risk individuals. By studying various CVD risk factors, models can be developed to predict an individual’s probability of developing a CVD. This aids in identifying individuals who require closer monitoring and preventive measures, and they can then be provided with appropriate medical interventions and health management recommendations. Identifying the risks of developing CVDs at an early stage offers more opportunities for treatment [9]. For example, targeted lifestyle changes such as adopting a healthy diet, engaging in moderate exercise, and reducing work-related stress can be implemented for individuals identified as high-risk. Additionally, pharmacological treatments to reduce the incidence of cardiovascular events may be considered. These early intervention measures contribute to reducing the incidence and severity of CVD. Predicting CVD also helps raise public health awareness, enabling individuals to better understand and evaluate their own cardiovascular health status. This could encourage people to proactively adopt healthy lifestyles and seek timely medical help and advice. Predicting CVDs also assists healthcare professionals in allocating and managing patients more effectively with limited resources. Predictive models can help healthcare institutions anticipate demand in advance and develop preventive measures and treatment plans, thereby reducing hospitalization times and medical costs and improving the efficiency of healthcare resource utilization. Simultaneously, the early identification of high-risk individuals and effective intervention measures can help alleviate the pressures on healthcare systems and reduce long-term healthcare costs.

However, inaccurate prediction results can also lead to overdiagnosis and overtreatment [10]. A prediction wrongly identifying an individual as being at high risk when they may actually have a low risk of developing a CVD can result in unnecessary medical interventions, the wastage of resources, and an increased psychological burden for the patient. On the other hand, if the prediction fails to accurately identify individuals who are actually at risk of developing a CVD, they may miss the opportunity for timely intervention and treatment, thus missing the chance to prevent disease progression. Furthermore, when the accuracy of the prediction results is low, people may doubt the effectiveness of the prediction and even develop mistrust towards the entire predictive model. This can lead to public disregard for prediction results and reduce their willingness to take subsequent action based on the predictions. Therefore, improving the accuracy of CVD predictions is an important research direction.

Currently, numerous experts and scholars are conducting predictive research on cardiovascular diseases. Arroyo and Delima (2022) [11] have enhanced cardiovascular disease prediction using a genetic algorithm (GA) to optimize artificial neural networks (ANN), improving their accuracy by 5.08%. Kim (2021) [12] utilized smartwatch data from the Korea National Health and Nutrition Examination Survey to predict cardiovascular disease prevalence. The support vector machine model achieved the highest accuracy, offering crucial insights for early and accurate diagnosis. Khan et al. (2023) [13] employed machine learning algorithms, including random forest, for the accurate prediction of cardiovascular disease (CVD). The random forest algorithm showed the highest accuracy (85.01%) and sensitivity (92.11%) among various methods tested. Moon et al. (2023) [14] used a literature embedding model and machine learning to predict cardiovascular disease (CVD) susceptibility accurately. With 96% accuracy, the model identifies related factors and genes, improving CVD prediction.

The above-mentioned literature discusses the accuracy of cardiovascular disease prediction; however, it does not explore techniques for consistently achieving high accuracy with limited resources. This study develops a method that significantly reduces the computational resources needed for accurate cardiovascular disease (CVD) prediction. The goal is to enhance early detection accessibility, revolutionizing cardiovascular health monitoring. By meeting the demands of point-of-care testing (POCT) with a resource-efficient artificial neural network (ANN) model, this approach enables the more precise prediction of individual CVD risks. This advancement allows for timely interventions, ultimately improving cardiovascular health and quality of life.

## 2. Materials and Methods

### 2.1. Mathematical Background

#### 2.1.1. Artificial Neural Network (ANN)

**Figure 1:**
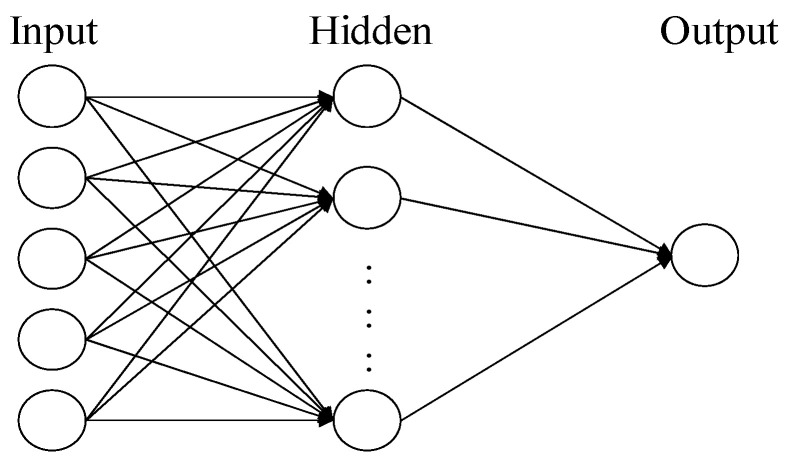
The architecture of A three-layer neural network.

The artificial neural network (ANN) is a fundamental machine learning model. Its structure comprises multiple neurons (or nodes), in which each neuron is connected to all neurons in the previous layer, forming a fully connected network [15]. In this structure, each neuron receives inputs from all the neurons in the previous layer and generates an output [16]. Such a design enables neural networks to learn complex nonlinear relationships. An artificial neural network consists of the following components [17,18]:

(1) Input Layer: the first layer of the neural network, responsible for receiving raw data or features.
(2) Hidden Layers: layers located between the input and output layers, responsible for further feature extraction and learning representations.
(3) Output Layer: the final layer of the neural network, responsible for generating the ultimate output. Figure 1 shows an ANN comprised of three layers of neurons.

(4) Weights and Biases: The strength of the connections between neurons is represented by weights. Each neuron also has a bias that affects its activation state.
(5) Activation Function: A function that transforms the weighted sum of neuron inputs into an output, introducing nonlinearity. Common activation functions include logistic, tanh, and ReLU (Rectified Linear Unit), which are explained as follows:
  (i) Logistic function (sigmoid function): The logistic function maps inputs to a range between (0, 1), and its output represents probability values. It is commonly used in binary classification problems. However, when inputs are significantly far from the origin (too large or too small), the gradient of the function becomes very small. This issue can lead to the problem of vanishing gradients, making it challenging to train neural networks effectively.
    (1) $$\sigma \left(x\right)=\frac{1}{1+e^{-x}}$$
  (ii) Tanh function: The tanh function maps inputs to the range (−1, 1). Compared to the logistic function, its output is more symmetric around the origin. Although it still faces the vanishing gradient problem, the tanh function’s outputs have an average closer to zero than the logistic function. This property helps mitigate the issue of vanishing gradients.
    (2) $$tanh\left(x\right)=\frac{e^{x}-e^{-x}1}{e^{x}+e^{-x}}$$
  (iii) ReLU function (Rectified Linear Unit): The ReLU function outputs the input value when it is positive and outputs zero when it is negative. It is a straightforward activation function, often leading to faster convergence during training. Unlike Sigmoid and tanh functions, ReLU does not suffer from the vanishing gradient problem.
    ReLU(*x*) = max(0,*x*) (3)
(6) Loss Function: Used to measure the disparity between the model’s predicted output and the actual output, the loss function is a critical aspect of the training process. Common loss functions include Mean Squared Error and Cross-Entropy.
(7) Optimizer: Responsible for updating the weights and biases of the neural network based on the gradients of the loss function, optimizers play a key role in the training process. Popular optimizers include lbfgs, sgd (stochastic gradient descent), and Adam.
  (i) lbfgs (limited-memory broyden-fletcher-goldfarb-shanno): lbfgs combines the benefits of gradient descent with the advantages of quasi-Newton methods, allowing for efficient convergence to the optimal solution.
  (ii) SGD (stochastic gradient descent): SGD is a fundamental optimization algorithm used in training artificial neural networks. Unlike traditional gradient descent, which computes gradients using the entire dataset, SGD updates the model’s parameters using only a single data point (or a small batch of data points) at a time. This stochastic nature introduces randomness into the optimization process.
  (iii) Adam (adaptive moment estimation): Adam is an adaptive learning rate optimization algorithm that combines the ideas of both momentum and RMSprop. Adam dynamically adjusts the learning rates based on the magnitude of the past gradients, providing faster convergence and better performance compared to fixed learning rate methods like traditional gradient descent.

Additionally, in an artificial neural network (ANN), there are two crucial hyperparameters: the learning rate and momentum rate, both of which have a significant impact on the performance of the ANN model. The learning rate determines the magnitude of the adjustments made to the model parameters during each step of optimization. If the learning rate is too large, the model may oscillate around the minimum and fail to converge. On the other hand, if it is too small, the model may converge very slowly or even get stuck in local minima [19,20]. As for the momentum rate, it introduces information from past gradients, helping to smooth the adjustment process of the weights. This prevents drastic fluctuations in weights during training, leading to a more stable learning process. Additionally, the momentum rate assists the model in escaping local minima, making it more likely to find the global minimum. This characteristic is particularly important in high-dimensional optimization problems, where local minima are more prevalent.

The artificial neural network involves two main steps: Forward Propagation and Backpropagation. Here is a detailed explanation including all relevant formulas:

Step 1. Forward Propagation:

(i) Linear Combination:
  For the l-th neural layer, compute the linear combination output $z^{\left(l\right)}$:
  (4) $$z^{\left(l\right)}=W^{\left(l\right)}a^{\left(l-1\right)}+b^{\left(l\right)}$$
  Here, $W^{\left(l\right)}$ represents the weight matrix of the l-th layer, $a^{\left(l-1\right)}$ is the activation output from the (l−1)-th layer, and $b^{\left(l\right)}$ is the boas of the l-th layer.
(ii) Activation Function:
  Apply the activation function σ to obtain the output $a^{\left(l\right)}$ for the l-th layer:
  (5) $$a^{\left(l\right)}=\sigma \left(z^{\left(l\right)}\right)$$
  Common activation functions include Sigmoid, ReLU, tanh, and Softmax.
(iii) Repeat Steps (i) and (iii):
  Iterate the linear combination and activation process until the output layer is reached.
(iv) Activation Function for the Output Layer.

Step 2. Backpropagation:

(i) Compute Loss:
  Utilize the loss function to calculate the error between the predicted and actual values. Common loss functions include Mean Squared Error (MSE) or Cross-Entropy, as follows:
  Mean Squared Error (MSE) (used in regression problems):
  (6) $$MSE=\frac{1}{n}\sum_{i=1}^{n}{\left(y_{i}-{\hat{y}}_{i}\right)}^{2}$$
  Cross-Entropy (used in classification problems):
  (7) $$\mathrm{Cross}-\mathrm{Entropy}=-\frac{1}{n}\sum_{i=1}^{n}y_{i}log\left({\hat{y}}_{i}\right)$$
  where $y_{i}$ represents the actual values, and ${\hat{y}}_{i}$ represents the predicted values.
(ii) Compute Gradients:
  Calculate the gradients of the loss with respect to weights $W^{\left(l\right)}$ and biases $b^{\left(l\right)}$, typically through partial differentiation, as follows:
  $$\frac{\partial Loss}{\partial W^{\left(l\right)}}\;\mathrm{and}\;\frac{\partial Loss}{\partial b^{\left(l\right)}}$$
(iii) Gradient Descent:
  Utilize the gradient descent to update weights and biases, aiming to minimize the loss function. The update rules are as follows:
  (8) $$W^{\left(l\right)}=W^{\left(l\right)}-\alpha \cdot \frac{\partial Loss}{\partial W^{\left(l\right)}}$$
  (9) $$b^{\left(l\right)}=b^{\left(l\right)}-\alpha \cdot \frac{\partial Loss}{\partial b^{\left(l\right)}}$$
  Here, α represents the learning rate.
(iv) Repeat Steps (i) to (iii):
  Iterate the process of computing loss, gradients, and gradient descent until the network’s parameters converge to optimal values.

There have been numerous studies using ANNs for modeling and prediction. Malhotra et al. (2022) utilized deep neural models in image segmentation tasks, specifically focusing on medical image datasets, evaluation metrics, and the performance of CNN-based networks [21]. Pantic et al. (2022) investigated the potential of ANNs in toxicology research, specifically for their ability to predict toxicity and classify chemical compounds based on their toxic effects, highlighting recent studies that demonstrated the scientific value of ANNs in evaluating and predicting the toxicity of compounds [22]. He et al. (2022), used an ANN algorithm to develop a lung cancer recognition model, which included determining the lesion area and employing an image segmentation algorithm to isolate and visualize the lung cancer lesion area, followed by a comparison experiment to validate the model’s accuracy [23]. Poradzka et al. (2023) adopted an ANN as a reliable prognostic method in diabetic foot syndrome (DFS) ulcers to aid in predicting the course and the outcome of treatment, particularly in identifying non-healing individuals [24]. Krasteva et al. (2023) [25] optimized DenseNet-3@128-32-4 with 137 features, ensuring the accurate classification of rhythms, including AF.

#### 2.1.2. Taguchi Method

The Taguchi method was created in 1950 by Dr. Genichi Taguchi, a Japanese expert. It gained rapid popularity in Japan and received significant attention from international quality professionals, leading to its recognition as the Taguchi method in the 1980s in the European and American quality management communities [26]. Through research work conducted in the 1950s and early 1960s, Dr. Taguchi developed the theory of robust design and achieved the successful development of many new products [27,28]. Additionally, by integrating technology and statistical methods, Dr. Taguchi enabled the attainment of optimal conditions in product design and manufacturing processes, leading to rapid improvements in cost and quality.

Traditional experimental design methods typically focus only on controllable factors, neglecting uncontrollable noise factors such as climate variations or inherent instrument instability. The application of the Taguchi method aims to eliminate effects caused by various factors, including the incorporation of noise factors in the experimental environment and actively identifying the optimal parameter settings. Moreover, Taguchi suggested employing orthogonal arrays in experimentation, which offers several benefits. First, it enables a reduction in the number of experiments required while still providing comprehensive and dependable information. Additionally, the use of orthogonal arrays ensures experimental reproducibility, enhancing the reliability of the results obtained.

In full factorial designs, as the number of variables increases, the number of required experiments also increases, which can lead to the increased complexity of the experimental approach. Taguchi proposed the use of orthogonal arrays in experimentation due to their advantageous features. By employing main-effects orthogonal arrays, researchers can achieve comprehensive and reliable experimental data while minimizing the number of required experiments. Furthermore, the use of orthogonal arrays ensures experimental reproducibility, thereby enhancing the reliability and validity of the obtained results. The symbols used in orthogonal arrays are explained as follows:

$$L_{a}(b^{c})$$

where *L* is the first letter of the Latin square, a is the overall number of the experiments conducted (rows), *b* is the number of levels assigned to the experimental settings, and *c* is a count of parameters that can be arranged (columns).

Table 1 shows an *L*_9_(3^4^) Taguchi orthogonal array. In the orthogonal array, A to D represent different hyperparameters, and the numbers 1, 2, and 3 within the table indicate levels of hyperparameter settings: levels 1, 2, and 3, respectively. Each column in the *L*_9_(3^4^) orthogonal array represents a variation in the setting of a specific hyperparameter for the experiments. The *L*_9_(3^4^) orthogonal array has a total of four columns, indicating that it can accommodate up to four hyperparameters. The rows correspond to the number of experiments in the orthogonal array, so the *L*_9_(3^4^) orthogonal array has a total of nine experiments.

In addition, to determine the impacts of parameters on product quality, Dr. Genichi Taguchi adopted the concept of signal-to-noise (SN) ratio from the telecommunications industry, which is measured in decibels (dB) [26]. By calculating the SN ratio, it is possible to identify which attributes have the greatest influence on product quality during the production process, thus optimizing the manufacturing process. A higher SN ratio indicates a more stable production process and better product quality, while a lower SN ratio indicates an unstable production process that requires measures for improvement. There are three different types of SN ratios based on different quality requirements: nominal-the-better (NTB), smaller-the-better (STB), and larger-the-better (LTB), as shown in Formulas (10)–(12) [29]. In these formulas, $\bar{y}$ represents the average value of each set of treatments, *m* is the target value for quality, *S*^2^ is the variance of each set of treatments, *yi* is the value of the treatment *i*, and *n* is the number of treatments.

(a) NTB refers to a type of SN ratio in which a precise target value is established, and the greater the proximity of the quality characteristic value to the target value, the more desirable the outcome. The ultimate objective of the quality characteristic is to attain the target value, representing the optimal functionality. The formula used for NTB is as follows:
  (10) $${\mathrm{SN}}_{\mathrm{NTB}}=-10\log\left[\frac{\sum_{i=1}^{n}(y_{i}-m{)}^{2}}{n}\right]=-10\log\left[{{\left(\bar{y}-m\right)}^{2}+S}^{2}\right].$$
(b) STB is an additional SN ratio category that aims for lower values of the quality characteristic. In STB, the ideal value for the quality characteristic is zero, representing the optimal condition. The formula used for STB is as follows:
  (11) $${SN}_{STB}=-10\log\frac{\sum_{i=1}^{n}y_{i}^{2}}{n}=-10\log({\bar{y}}^{2}+S^{2}).$$
(c) LTB is another SN ratio type that prioritizes higher values for the quality characteristic. In LTB, the ideal functionality for the quality characteristic is considered infinite, indicating the most desirable outcome. The formula used for LTB is as follows:
  (12) $${SN}_{LTB}=-10\log\frac{\sum_{i=1}^{n}\frac{1}{y_{i}^{2}}}{n}.$$

Currently, many studies use the Taguchi method for the optimal improvement of engineering problems. Kaziz et al. (2023) conducted an *L*_8_(2^5^) Taguchi orthogonal array and analysis of variance to minimize biosensor detection times under an alternating current electrothermal force [30]. Tseng et al. (2022) used the Taguchi method with an indigenous polymethyl methacrylate (PMMA) slit gauge to optimize the image quality of brain gray and white matter [31]. Safaei et al. (2022) applied the Taguchi method to test the antimicrobial properties of an alginate/zirconia bionanocomposite [32]. Lagzian et al. (2022) used the Taguchi method and a SN ratio to identify key characteristics and optimize their agent-based model for the accurate analysis of cancer stem cells and tumor growth, with migration, tumor location, and cell senescence identified as the most important features [33].

#### 2.1.3. Analysis of Variance (ANOVA)

When researchers want to consider multiple categorical independent variables and test differences between multiple groups’ means, they need to use an ANOVA [34]. If these independent variables are categorical variables, and the dependent variable is a continuous variable, statistical analysis is required to handle the relationships between multiple groups’ means. In other words, the variation in the dependent variable may be influenced by different levels of the independent variables. This study used a two-factor experimental design as an example, as shown in Table 2.

In a multiple-factor design, the testing of means requires the use of an ANOVA to compare variations in different means. In Table 2, there are two independent variables, namely factors A and B. Factor A has two levels, A_1_ and A_2_, while factor B has three levels, B_1_, B_2_, and B_3_. The effects of these two independent variables on the dependent variable must be examined using a two-factor ANOVA. The difference in means for factor A is referred to as the “main effect of A”, while the difference in means for factor B is referred to as the “main effect of B”. The significance of these two effects can be determined through *F*-tests. If the *F*-test demonstrates a notable disparity in means across each level of factor A or factor B, it signifies a substantial impact of factor A or factor B on the dependent variable.

A two-factor ANOVA is used to compare two group means. Each mean is calculated from a set of small samples representing a population parameter. Therefore, the two-factor ANOVA is a hypothesis test for multiple populations. Variability in the dependent variable across all observations is represented by the “total sum of squares” (SST), which is calculated by subtracting each observation’s raw data from the overall mean and summing the squared differences. Variability of the raw data can be partitioned into the “between-group effect SSF” (differences in the dependent variable caused by the grouping of levels in the independent variables) and the “within-group effect SSE”, which represents the random error in the response. The between-group effect can be further divided into contributions from factors A and B, as shown in the following Equation (13) [34]:

SST = SSF + SSE = (SSA + SSB) + SSE.(13)

Each sum of squares (SS) corresponds to specific degrees of freedom (DF). When the number of observations is *M*, the degrees of freedom for SST are *M* − 1. For the dependent variable with a levels for *F*, the degrees of freedom for SSF are *a* − 1. Finally, the degrees of freedom for the error are *M* − *a*. Dividing the SS by its corresponding DF provides a reliable source of variation assessment, known as the mean of squares (MS). In a multiple-factor ANOVA, the *F*-value is obtained by dividing the mean of squares (MS) of the variability within each factor’s levels by the Mean Squared Error (MSE). If the probability value associated with the *F*-value exceeds the significance level (in this study, α = 0.1), then the effect is significant.

Currently, many researchers use ANOVAs to assess their studies. Blanco-Topping (2021) analyzed patient satisfaction, measured by the HCAHPS survey, across different years in the Maryland Global Payment implementation cycle using a one-way ANOVA of nine variables [35]. Mahesh and Kandasamy (2023) used the ANOVA method to investigate the impacts of drilling parameters (feed, speed, and drill diameter) on the delamination and taperness of the hole in hybrid glass fiber-reinforced plastic (GFRP)/Al_2_O_3_ [36]. Adesegun et al. (2020) employed an ANOVA to assess the knowledge, attitudes, and practices related to coronavirus disease 2019 (COVID-19) among the Nigerian public [37].

### 2.2. Methodological Design

In the process of improving model accuracy, many researchers often rely on empirical knowledge to select important levels of model hyperparameters for experimentation. However, the improvement in model accuracy is often not significant, resulting in a waste of modeling resources. If we first identify the trend of each model hyperparameter’s settings in the process of improving the accuracy and then optimize and adjust each model hyperparameter accordingly, we can more efficiently improve model accuracy.

The Taguchi method differs from trial-and-error methods or one-factor-at-a-time experiments. It allows for the consideration of multiple model hyperparameters at different levels to assess their impacts on the accuracy of a CVD prediction model with the fewest number of modeling experiments. In addition to reducing the number of modeling experiments, the Taguchi method can also identify optimal trends in model hyperparameter settings. For example, in ANNs, a higher value for the momentum rate might lead to improved accuracy. To effectively enhance the accuracy of CVD prediction models, this study proposes a two-stage Taguchi optimization (TSTO) method to identify the best model hyperparameter setting for the ANN model.

**Figure 2:**
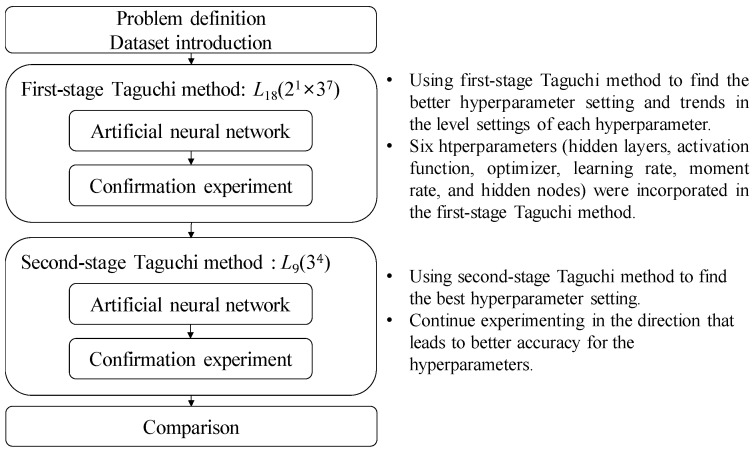
Proposed methodology.

The analytical process in this study can be divided into four steps, as shown in Figure 2.

(1) Problem definition: describing the source of the dataset and its relevant feature data.
(2) Using the first-stage Taguchi method to find improved hyperparameter settings of the ANN model and trends in the level setting of each hyperparameter: This step involves using the Taguchi method, specifically *L*_18_(2^1^ × 3^7^), to collect model accuracy data for various hyperparameter settings. In this study, a ANN was used as the predictive model for CVDs. Experimental, analytical techniques such as an orthogonal array, parameter response table, parameter response graph, and ANOVA were then employed to assist in identifying better settings for the hyperparameter levels and the preferred trend for each hyperparameter that affected the average accuracy of CVD predictions. Finally, five confirmation experiments were conducted on the identified improved hyperparameter settings to ensure reproducibility.
(3) Using the second-stage Taguchi method to find the best hyperparameter settings of the ANN model: In this step, another Taguchi method, specifically *L*_9_(3^4^), was once again used to collect experimental data. The purpose was to conduct a second round of the Taguchi method within the hyperparameter range that may contain the best solution. Through the analytical techniques mentioned earlier, including the parameter response table, parameter response graph, and ANOVA, the best settings of the hyperparameter levels were determined. After identifying the best hyperparameter settings, a set of five confirmation experiments was conducted to validate the efficacy of the proposed methodology.
(4) Comparison: The best model accuracy obtained from the two-stage Taguchi optimization method was compared to the accuracy of relevant models reported in the literature to confirm the improvement achieved in this study.

## 3. Results

### 3.1. Dataset Introduction

We used the publicly accessible Kaggle Cardiovascular Disease dataset obtained from the source referenced as [38]. The dataset consists of 70,000 instances gathered from medical examinations. The dataset consists of 12 variables, where variables 1 to 11 represent input features, and variable 12 represents the output feature. To facilitate the training and testing of our ANNs, we divided the dataset into 80% of instances for training and 20% for testing purposes. The descriptions for the dataset features are presented in Table 3 [38], and provided below.

(a) “Age” is an integer variable measured in days. Analyzing the age distribution revealed that there were 8159 individuals (11.66%) below 16,000 days old, 10,027 individuals (14.32%) between 16,000 and 17,999 days old, 20,490 individuals (29.27%) between 18,000 and 19,999 days old, 20,011 individuals (28.59%) between 20,000 and 21,999 days old, and 11,313 individuals (16.16%) between 22,000 and 24,000 days old.
(b) “Height” is an integer variable measured in centimeters. Observing the height distribution revealed that 1537 individuals (2.20%) were below 150 cm, 16,986 individuals (24.27%) were between 150 and 159 cm, 33,463 individuals (47.80%) were between 160 and 169 cm, 15,696 individuals (22.42%) were between 170 and 179 cm, 2213 individuals (3.16%) were between 180 and 189 cm, and 105 individuals (0.15%) were above 190 cm.
(c) “Weight” is a float variable measured in kilograms. Analyzing the weight distribution revealed that 987 individuals (1.41%) weighed less than 50 kg, 7174 individuals (10.25%) weighed between 50 and 59 kg, 20,690 individuals (29.56%) weighed between 60 and 69 kg, 19,476 individuals (27.82%) weighed between 70 and 79 kg, 11,989 individuals (17.13%) weighed between 80 and 89 kg, 5831 individuals (8.33%) weighed between 90 and 99 kg, and 3853 individuals (5.50%) weighed over 100 kg.
(d) “Gender” is a categorical variable represented as follows: 1 for female and 2 for male. Out of 70,000 patients, 45,530 were female (approximately 65.4%) and 24,470 male (approximately 34.96%).
(e) “Systolic blood pressure” is an integer variable. Observing its distribution revealed that there were 13,038 individuals (18.63%) with systolic pressure below 120, 37,561 individuals (53.66%) with systolic pressure between 120 and 139, 14,436 individuals (20.62%) with systolic pressure between 140 and 159, 3901 individuals (5.57%) with systolic pressure between 160 and 179, and 1064 individuals (1.52%) with systolic pressure above 180.
(f) “Diastolic blood pressure” is also an integer variable. Analyzing its distribution revealed that 14,116 individuals (20.17%) had diastolic pressure below 80, 35,450 individuals (50.64%) had diastolic pressure between 80 and 89, 14,612 individuals (20.87%) had diastolic pressure between 90 and 99, 4139 individuals (5.91%) had diastolic pressure between 100 and 109, and 1683 individuals (2.40%) had diastolic pressure above 110.
(g) “Cholesterol” is a categorical variable, represented as follows: 1 for normal, 2 for above normal, and 3 for well above normal. Out of 70,000 patients, 52,385 (approximately 74.84%) had normal cholesterol levels, 9549 (approximately 13.64%) had above normal levels, and 8066 (approximately 11.52%) had well above normal levels.
(h) “Glucose” is a categorical variable, represented by 1 for normal, 2 for above normal, and 3 for well above normal. Out of 70,000 patients, 59,479 individuals (approximately 84.97%) had normal glucose levels, 5190 individuals (approximately 7.41%) had above normal levels, and 5331 individuals (approximately 7.62%) had well above normal levels.
(i) “Smoking” is a binary variable, with 0 indicating non-smokers and 1 indicating smokers. Of the 70,000 patients, 63,831 individuals (approximately 91.19%) were non-smokers, and 6169 individuals (approximately 8.81%) were smokers.
(j) “Alcohol intake” is a binary variable, with 0 indicating no alcohol consumption and 1 indicating alcohol consumption. Out of 70,000 patients, 66,236 individuals (approximately 94.62%) did not consume alcohol, and 3764 individuals (approximately 5.38%) consumed alcohol.
(k) “Physical activity” is a binary variable, with 0 indicating no physical activity and 1 indicating physical activity. Out of 70,000 patients, 13,739 individuals (approximately 19.63%) were inactive, and 56,261 individuals (approximately 80.37%) were physically active.
(l) The “Presence (or absence) of cardiovascular disease” is a binary variable, with 0 indicating the absence of cardiovascular disease and 1 indicating its presence. Out of 70,000 patients, 35,021 individuals (approximately 50.03%) did not have cardiovascular disease, while 34,979 individuals (approximately 49.97%) had cardiovascular disease.

Given the diverse input features, methods to establish techniques for their normalization are crucial, as normalization typically significantly influences accuracy. In this study, we adopt z-score normalization for input features, ensuring standardized scaling and robustness in our analytical assessments. This approach enhances accuracy and maintains consistency across diverse input features.

### 3.2. Using the First-Stage Taguchi Method to Find Better Hyperparameter Settings

To improve the predictive accuracy of the prediction model, this study selected six model hyperparameters that may affect the ANN. These six model hyperparameters were the hidden layers, activation function, optimizer, learning rate, moment rate, and hidden nodes. Table 4 shows the experimental configurations of the model hyperparameters, with level 1 denoting the low setting level and level 2 representing the high setting level. The Taguchi method, specifically *L*_18_(2^1^ × 3^7^), was chosen to conduct the first stage of the Taguchi method to find better hyperparameter settings of the ANN model and trends in the level setting of each model hyperparameter. The model hyperparameters X_1_~X_6_ were arranged in columns 2 to 7 of the *L*_18_(2^1^ × 3^7^) orthogonal array.

The Taguchi design incorporates the concept of noise to address the randomness issue during ANN training. By enhancing the signal-to-noise ratio, it effectively reduces the random variations in model training, ensuring consistent performance across each training. If the grid search method is used to find better settings for the six model hyperparameters, each with three levels and repeated three times, a total of 3^6^ × 3 = 2187 experiments would be required. However, in the first-stage Taguchi method, this study only conducted a total of 18 × 3 = 54 experiments with different model hyperparameter settings. Due to the limited number of experiments conducted in this case, high-end hardware configuration or the use of a graphics processing unit (GPU) was not required. The hardware for the ultra-low-cost personal device used in this study is described here:

(a) central processing unit: 11th Generation Intel(R) Core(TM) i5-1135G7 @ 2.40 GHz;
(b) random access memory: 8.00 GB;
(c) 64-bit system; and
(d) Python version: 3.9.13.

In *L*_18_(2^1^ × 3^7^), the Taguchi orthogonal array with 54 runs took a total of 33.92 min to compute on a personal computer, averaging approximately 0.628 min per run. When discussing the grid search of 2187 runs, since this study did not actually complete all 2187 runs, estimating an average time of 0.628 min per run suggests that the full grid search of 2187 runs would potentially require approximately 1373.775 min.

We analyzed the effects of hyperparameters to identify crucial ones and observe trends in the level settings of each hyperparameter. The effect of a factor is defined as the maximum difference in the overall average accuracy across various levels. The hyperparameter response table and response graph show the analysis results for all the hyperparameter effects.

**Figure 3:**
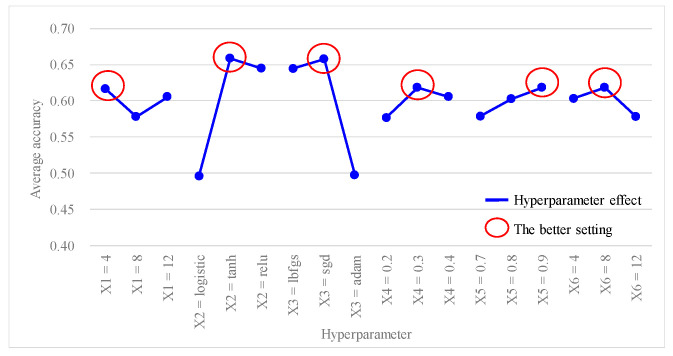
Hyperparameter response graph of average accuracy for *L*_18_(2^1^ × 3^7^).

Table 5 shows the predictive accuracy obtained from each experiment using the *L*_18_(2^1^ × 3^7^) Taguchi orthogonal array. Each experiment was repeated three times for columns N_1_, N_2_, and N_3_. Table 6 shows the findings of the hyperparameter response table, while Figure 3 shows the hyperparameter response graph for the average accuracy of the ANN model. Additionally, Table 7 shows the results of the ANOVA table pertaining to the average accuracy. Table 6 shows the ranking of hyperparameter effects on the average accuracy of the ANN model in descending order: X_2_ (0.1619) > X_3_ (0.1605) > X_6_ (0.0408) > X_4_ (0.0406) > X_5_ (0.04) > X_1_ (0.0389). Table 7 shows that *p* values for hyperparameters X_2_ and X_3_ were both <0.1, indicating that these two hyperparameters significantly contributed to the average accuracy of the ANN model; they were important factors influencing the predictive accuracy. In Figure 3, better average accuracy settings of the hyperparameters were determined as follows: X_1_ (hidden layers) = 4; X_2_ (activation function) = tanh; X_3_ (optimizer) = sgd; X_4_ (learning rate) = 0.3; X_5_ (moment rate) = 0.9; and X_6_ (hidden nodes) = 8. In this case, we also found that increasing the number of hidden layers and nodes in the hidden layers did not necessarily result in higher accuracy.

**Figure 4:**
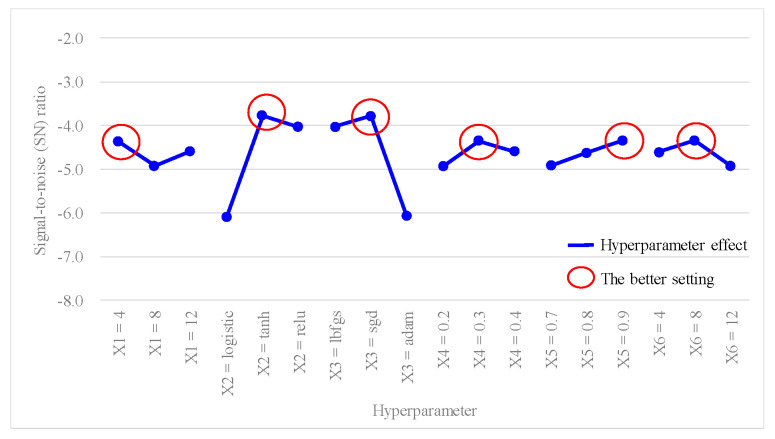
Hyperparameter response graph of the signal-to-noise (SN) ratio for *L*_18_(2^1^ × 3^7^).

Furthermore, since the average accuracy of the ANN model in this study was considered as a larger-the-better (LTB) type, the SN ratio for each experiment was calculated using Equation (12), and results are presented in the last column of Table 5. Table 8 shows the outcomes of the hyperparameter response table, while Figure 4 shows the hyperparameter response graph for the SN ratio of the ANN model. Additionally, Table 9 provides the results of the ANOVA table relating to the SN ratio. Table 8 shows the ranking of the hyperparameter effects on the SN ratio of the ANN model in descending order: X_2_ (2.3102) > X_3_ (2.2924) > X_6_ (0.5760) > X_4_ (0.5754) > X_5_ (0.5723) > X_1_ (0.5562). Table 9 shows that *p* values for hyperparameters X_2_ and X_3_ were both < 0.1, indicating that these two hyperparameters significantly contributed to the SN ratio of the ANN model. They were important factors influencing the SN ratio. In Figure 4, the better SN ratio setting the of hyperparameters was determined as follows: X_1_ (hidden layers) = 4, X_2_ (activation function) = tanh, X_3_ (optimizer) = sgd, X_4_ (learning rate) = 0.3, X_5_ (moment rate) = 0.9, and X_6_ (hidden nodes) = 8.

Based on the previous analysis of the average accuracy and SN ratio, better settings for the hyperparameter levels were determined as follows: X_1_ (hidden layers) = 4, X_2_ (activation function) = tanh, X_3_ (optimizer) = sgd, X_4_ (learning rate) = 0.3, X_5_ (moment rate) = 0.9, and X_6_ (hidden nodes) = 8. Since the better setting for X_1_ (hidden layer) was at level 1, it was represented as X_1_(1). The improved settings of the model hyperparameters can also be expressed as X_1_(1), X_2_(2), X_3_(2), X_4_(2), X_5_(3) and X_6_(2). To predict the average accuracy and SN ratio using the improved settings of the model hyperparameter levels, this study applied Equation (14), as recommended by Taguchi (1986) [27]:

(14) $$Predicted\;value=Mean+\left[X_{1}\left(1\right)-Mean\right]+\left[X_{2}\left(2\right)-Mean\right]+\left[X_{3}\left(2\right)-Mean\right]+\left[X_{4}\left(2\right)-Mean\right]+\left[X_{5}\left(3\right)-Mean\right]+\left[X_{6}\left(2\right)-Mean\right].$$

The ANOVA results in Table 7 and Table 9 show that hyperparameters X_1_, X_4_, X_5,_ and X_6_ had minimal impacts on the average accuracy and SN ratio. Therefore, when predicting the average accuracy and SN ratio, these hyperparameters (X_1_, X_4_, X_5,_ and X_6_) were not considered. The predicted results for the accuracy and SN ratio were as Equation (15):

(15) $$\begin{array}{c} Accuracy=0.5999+\left[0.6587-0.5999\right]+\left[0.6578-0.5999\right]=0.7166\;and\;\\ SN\;ratio=-4.625+\left[-3.7673-\left(-4.625\right)\right]+\left[-3.7773-\left(-4.625\right)\right]=-2.919. \end{array}$$

To verify the improved settings of the model hyperparameters obtained from the first-stage *L*_18_(2^1^ × 3^7^) Taguchi method, this study conducted five confirmation experiments. The 95% confidence intervals (CIs) for the average accuracy and SN ratio were calculated using the following Equation (16) [26]:

(16) $$CI=\sqrt{F_{\alpha ;1,\nu_{2}}\times V_{e}\times \left(\frac{1}{n_{eff}}+\frac{1}{r}\right)};$$

where $n_{eff}=\frac{\mathrm{Total}\;\mathrm{number}\;\mathrm{of}\;\mathrm{experiment}\;s}{1+\left[\mathrm{Sum}\;\mathrm{of}\;\mathrm{degrees}\;\mathrm{of}\;\mathrm{freedom}\;\mathrm{for}\;\mathrm{factors}\;\mathrm{used}\;\mathrm{to}\;\mathrm{estimate}\;\mathrm{the}\;\mathrm{mean}\right]}$, $F_{\alpha ;1,\upsilon_{2}}$ is the *F*-value with a significant level α, *α* is the significance level, *ν*_2_ is the degree of freedom associated with the combined error variance, *V_e_* is the variance of the combined error, *n_eff_* is the effective sample size, and *r* is the number of samples for confirmation experiments.

The 95% CIs for the average accuracy and SN ratio were calculated as Equation (17):

(17) $$\begin{array}{c} n_{eff}=\frac{18}{1+\nu_{X_{2}}+\nu_{X_{3}}}=\frac{18}{1+2+2}=3.6;\\ \begin{array}{l} CI_{Mean\;of\;Accuracy}=\sqrt{F_{0.05;1,13}\times V_{e}\times \left(\frac{1}{n_{eff}}+\frac{1}{r}\right)}\\ \;\;\;\;\;\;=\sqrt{4.6672\times 0.0039\times \left(\frac{1}{3.6}+\frac{1}{5}\right)}=0.0933;\;and \end{array}\\ \begin{array}{l} CI_{SN}=\sqrt{F_{0.05;1,13}\times V_{e}\times \left(\frac{1}{n_{eff}}+\frac{1}{r}\right)}\\ \;\;=\sqrt{4.6672\times 0.815\times \left(\frac{1}{3.6}+\frac{1}{5}\right)}=1.3481 \end{array}. \end{array}$$

**Figure 5:**
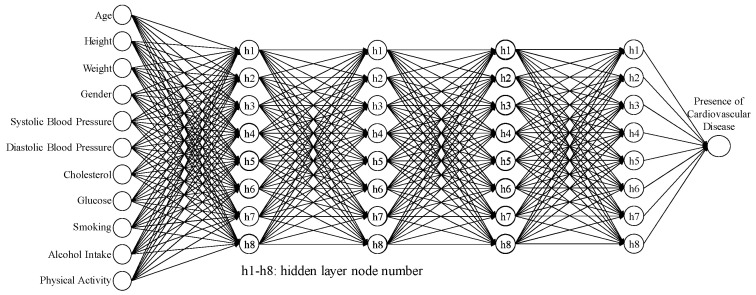
ANN architecture for four hidden layers and eight hidden nodes.

Therefore, the 95% CI for the average accuracy was (0.6233, 0.8099), and the 95% CI for the SN ratio was (−4.2671, −1.5709). Table 10 shows the results of five confirmation experiments; the average accuracy was 0.7383, and the SN ratio was −2.636 dB. Both of these values fall within their respective CIs, indicating the success of the confirmation experiments and the reproducibility of the improved model hyperparameter settings. The ANN model architecture identified through the first-stage Taguchi method includes four hidden layers and eight hidden nodes, as shown in Figure 5.

### 3.3. Using the Second-Stage Taguchi Method to Find the Best Hyperparameter Settings

From Figure 3 and Figure 4, it can be observed that there was a better average accuracy and SN ratio when X_1_ (hidden layers) was set to four hidden layers. To avoid further reducing the number of hidden layers and potentially decreasing the predictive accuracy, this study fixed X_1_ (hidden layers) at four layers. Additionally, since X_2_ (activation function) and X_3_ (optimizer) were categorical variables and were identified as important model hyperparameters through the ANOVA table, this study fixed X_2_ (activation function) as tanh and X_3_ (optimizer) as sgd. Furthermore, based on the analytical results from Figure 3 and Figure 4, setting X_4_ (learning rate) at 0.3, X_5_ (moment rate) at 0.9, and X_6_ (hidden nodes) at 8 resulted in a better average accuracy and SN ratio. Therefore, this study planned to set X_4_ (learning rate) to 0.3 ± 0.05, X_5_ (moment rate) to 0.9 ± 0.05, and X_6_ (hidden nodes) to 8 ± 2, and continued with the second-stage Taguchi method to find a further improved ANN model accuracy. The hyperparameters and their settings for this stage are shown in Table 11. In this stage, the Taguchi method used the *L*_9_(3^4^) orthogonal array, where the three model hyperparameters, X_4_ (learning rate), X_5_ (moment rate), and X_6_ (hidden nodes), were assigned to columns 1 to 3 of the *L*_9_(3^4^) orthogonal array, as shown in Table 12. Through the second-stage Taguchi orthogonal array *L*_9_(3^4^) with three repeats, relevant data containing the potential best solutions were collected, and the best settings of the model hyperparameters were identified. If a grid search method was applied to find the best setting for the three hyperparameters, each with three levels, and repeated three times, a total of 3^3^ × 3 = 81 experiments would be required. However, at this stage, a total of only 9 × 3 = 27 experiments were conducted for the Taguchi method.

**Figure 6:**
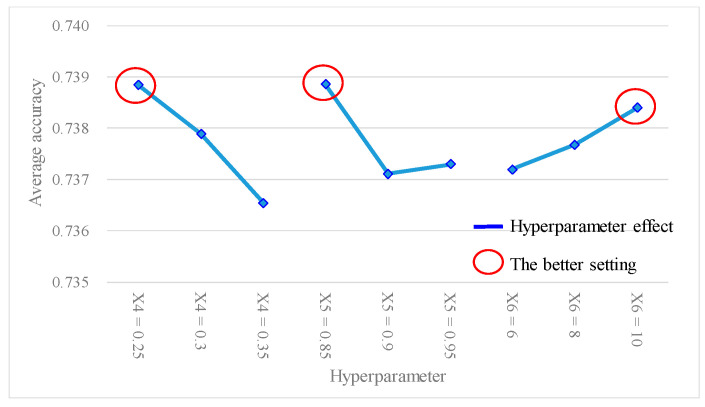
Hyperparameter response graph of average accuracy for *L*_9_(3^4^).

**Figure 7:**
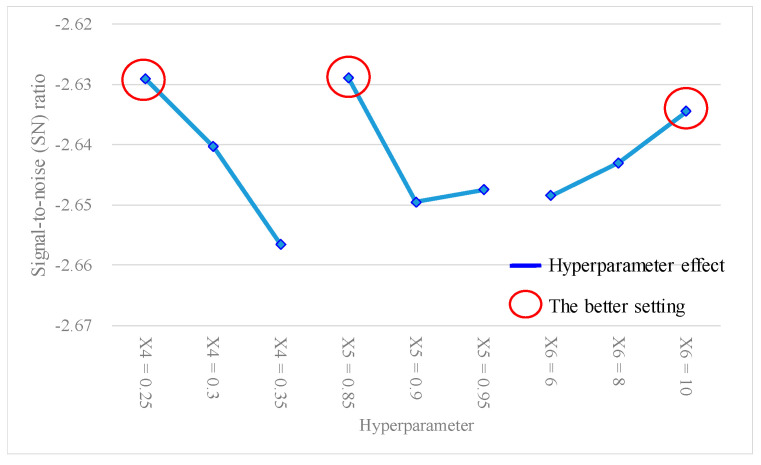
Hyperparameter response graph of the signal-to-noise (SN) ratio for *L*_9_(3^4^).

Table 12 shows the experimental results of the average accuracy and SN ratio of the ANN model under different experimental level settings, using the *L*_9_(3^4^) Taguchi method. Table 13 presents the findings of the hyperparameter response table, while Figure 6 gives the hyperparameter response graph for the analytical results of the average accuracy of the ANN model. Additionally, Table 14 provides the ANOVA table pertaining to the average accuracy. Table 13 shows the descending order ranking of the effects of each model hyperparameter on the ANN model average accuracy as follows: X_4_ (0.0023) > X_5_ (0.0017) > X_6_ (0.0012). Table 14 shows that the *p* values for hyperparameters X_4_ and X_5_ were both < 0.1, indicating that these two model hyperparameters significantly contributed to the average accuracy of the ANN model and were important factors affecting the accuracy. In Figure 6, the best settings of the hyperparameters were determined as X_4_ (learning rate) = 0.25, X_5_ (moment rate) = 0.85, and X_6_ (hidden nodes) = 10. Furthermore, Table 15 shows the results of the hyperparameter response table, Figure 7 shows the hyperparameter response graph, and Table 16 presents the ANOVA table for the analysis of the SN ratio in the ANN model. Table 15 shows the descending order ranking of the effects of each model hyperparameter on the ANN model’s SN ratio as follows: X_4_ (0.027) > X_5_ (0.021) > X_6_ (0.014). Table 16 demonstrates that the *p* values for model hyperparameters X_4_ and X_5_ were both <0.1, indicating their significant contributions to the SN ratio of the ANN model and their importance as influential hyperparameters. In Figure 7, the best settings of model hyperparameters were set as X_4_ (learning rate) = 0.25, X_5_ (moment rate) = 0.85, and X_6_ (hidden nodes) = 10.

Based on the analytical results of the average accuracy and SN ratio mentioned above, the best settings for the hyperparameter levels obtained from the second-stage Taguchi method were X_4_ (learning rate) = 0.25, X_5_ (moment rate) = 0.85, and X_6_ (hidden nodes) = 10. The best settings for the hyperparameter levels can also be expressed as X_4_(1), X_5_(1), and X_6_(3). This study used Equation (14) once again to predict the average accuracy and SN ratio using the best hyperparameter levels. According to the ANOVA results in Table 14 and Table 16, it is evident that hyperparameters such as X_4_ and X_5_ had a significant impact on the average accuracy and SN ratio. Therefore, when predicting the average accuracy and SN ratio, consideration was given to hyperparameters such as X_4_ and X_5_. The predicted results for the accuracy and SN ratio were as Equation (18):

(18) $$\begin{array}{c} Accuracy=0.7378+\left[0.7388-0.7378\right]+\left[0.7389-0.7378\right]=0.7399\;and\\ SNratio=-2.642+\left[-2.629-\left(-2.642\right)\right]+\left[-2.629-\left(-2.642\right)\right]=-2.616. \end{array}$$

To verify the best settings of the hyperparameters identified by the second-stage *L*_9_(3^4^) Taguchi orthogonal array, this study conducted five confirmation experiments. The 95% CIs for the average accuracy and SN ratio were calculated using Equation (16). The 95% CIs for the average accuracy and SN ratio were calculated as Equation (19):

(19) $$\begin{array}{c} n_{eff}=\frac{9}{1+\nu_{X_{4}}+\nu_{X_{5}}}=\frac{9}{1+2+2}=1.8;\\ \begin{array}{l} CI_{Mean\;of\;Accuracy}=\sqrt{F_{0.05;1,4}\times V_{e}\times \left(\frac{1}{n_{eff}}+\frac{1}{r}\right)}\\ \;\;\;\;\;\;\;=\sqrt{7.7086\times 0.000001\times \left(\frac{1}{1.8}+\frac{1}{5}\right)}=0.0024\;;\;and \end{array}\\ \begin{array}{l} CI_{SN}=\sqrt{F_{0.05;1,4}\times V_{e}\times \left(\frac{1}{n_{eff}}+\frac{1}{r}\right)}\\ \;\;=\sqrt{7.7086\times 0.000093\times \left(\frac{1}{1.8}+\frac{1}{5}\right)}=0.0233 \end{array} \end{array}$$

**Figure 8:**
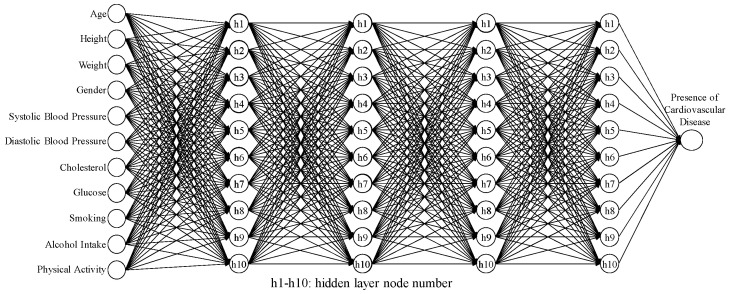
ANN architecture for four hidden layers and 10 hidden nodes.

The 95% CI for the average accuracy was (0.7375, 0.7423), and the 95% CI for the SN ratio was (−2.640, −2.593). The results of these five confirmations (as shown in Table 17) indicated that the average accuracy was 0.7414, and the SN ratio was −2.598 dB. Both values fell within their respective CIs, indicating successful confirmation experiments and the good reproducibility of the best hyperparameter settings. Furthermore, the second-stage Taguchi method showed better improvements in the hyperparameter settings compared to the first-stage Taguchi method. The comparison results are shown in Table 18. The average accuracy of the model increased from 0.7383 to 0.7414, an improvement of 0.0032. The standard deviation decreased from 0.0017 to 0.0015, an improvement of 0.00002. The SN ratio improved from −2.636 to −2.598 dB, an improvement of 0.0374 dB. Finally, the ANN model architecture identified through the second-stage Taguchi method includes four hidden layers and 10 hidden nodes, as shown in Figure 8.

### 3.4. Comparative Study

To compare differences in the predictive accuracies of the proposed methodology in this study and other machine learning algorithms using the same dataset, we compared it with the GA-ANN model proposed by Arroyo and Delima (2022) [11]. In their research, Arroyo and Delima also compared the GA-ANN with other predictive algorithms such as an ANN, logistic regression, decision tree, random forest, support vector machine, and K-nearest neighbor. We used the same dataset as Arroyo and Delima (2022), but we utilized the results directly from the referenced paper [11] without any additional processing. Table 19 compares the TSTO method proposed in this study with the GA-ANN method presented by Arroyo and Delima in 2022 in their work. From Table 19, it can be observed that the ANN optimized by the TSTO method achieved the highest accuracy among the different predictive algorithms used. The study demonstrated that the TSTO method effectively optimized the accuracy of the predictive model.

## 4. Discussion

Improving the accuracy of CVD prediction models has always been an important research focus. By continually improving predictive models, we can more accurately predict an individual’s risk of CVDs and provide opportunities for early interventions and treatment. In order to enhance the accuracy of the CVD prediction model, we proposed a TSTO method framework that enables the continuous approximation of the best hyperparameter settings for the ANN model.

In the first stage of this study, experiments were conducted using a reduced number of trials via the Taguchi method *L*_18_(2^1^ × 3^7^). Originally, conducting experiments with three levels for each of the six hyperparameters and repeating them three times would require 3^6^ × 3 = 2187 experiments. However, with the Taguchi orthogonal array *L*_18_(2^1^ × 3^7^), only 18 × 3 = 54 experiments were needed. It reduced the number of experiments by a factor of 40.5 compared to the grid search method. TSTO can significantly reduce computational resources, especially when the dimensionality of the dataset increases, or when the number of considered hyperparameters grows.

Results of the first-stage Taguchi method also determined the effects of the six hyperparameters in the following order: X_2_ (activation function) > X_3_ (optimizer) > X_6_ (hidden nodes) > X_4_ (learning rate) > X_5_ (moment rate) > X_1_ (hidden layers). Additionally, the hyperparameter response graph and ANOVA table confirmed better settings for the six hyperparameters as follows: X_1_ (hidden layers) = 4, X_2_ (activation function) = tanh, X_3_ (optimizer) = sgd, X_4_ (learning rate) = 0.3, X_5_ (moment rate) = 0.9, and X_6_ (hidden nodes) = 8. The first confirmation experiment was performed with these improved hyperparameter settings, resulting in an average accuracy of 0.7383.

In the second stage, the predicted accuracies of the three hyperparameters were collected using the Taguchi method *L*_9_(3^4^). Conducting experiments with three levels for each of the three hyperparameters and repeating them three times would require 3^3^ × 3 = 81 experiments. However, with the Taguchi orthogonal array *L*_9_(3^4^), only 9 × 3 = 27 experiments were needed. In this stage, the effects of the three hyperparameters were reconfirmed, with X_4_ (learning rate) > X_5_ (moment rate) > X_6_ (hidden nodes) in descending order. The best settings for the three hyperparameters were also determined through the hyperparameter response graph and ANOVA table, resulting in X_4_ (learning rate) = 0.25, X_5_ (moment rate) = 0.85, and X_6_ (hidden nodes) = 10. The best ANN architecture discovered in this stage includes four hidden layers and 10 hidden nodes. The second confirmation experiment was conducted with the best hyperparameter settings, resulting in an average accuracy of 0.7414 for the predictive model. Furthermore, the second-stage Taguchi method demonstrated improved effects compared to the hyperparameter settings obtained in the first stage. The average accuracy of the model increased from 0.7383 to 0.7414.

Finally, the proposed two-stage Taguchi method achieved higher accuracy than the state-of-the-art GA-ANN model for predicting CVD risk, which could further improve survival rates of cardiovascular patients. Moreover, the proposed method can significantly reduce the computational resources required for a ANN model. It can be easily combined with low-power computing devices or biosensors and further extended to individual users to achieve the goal of POCT.

Although this study achieved impressive accuracy, it still has several limitations. The current set of hyperparameter levels established in this study is discrete. In the future, a more extensive Taguchi method with multiple stages can be employed to approach the global optimal solution. Furthermore, the combinations of the hyperparameters identified in this study are specific to the cardiovascular disease (CVD) dataset. The proposed technique (TSTO) could be extended to other medical fields in future studies. Besides, this study utilized a single CVD dataset. In the future, independent CVD datasets from different healthcare organizations could be applied to validate the proposed TSTO technique.

## 5. Conclusions

This study enhances cardiovascular disease prediction using personal devices, aligning with point-of-care testing objectives. A two-stage Taguchi optimization (TSTO) method was introduced to boost the predictive accuracy of an ANN model while minimizing computational costs. The TSTO method is applied once during the training process and can run on any platform. TSTO does not impose constraints; instead, it simplifies the network architecture. It incorporates optimal hyperparameter settings established during the design phase, some of which may be redundant. The resulting final model can be easily combined with low-power computing devices or biosensors and extended to individual users to achieve the goal of POCT.

## Figures and Tables

**Table 1:** *L*_9_(3^4^) orthogonal array.

No	A	B	C	D
1	1	1	1	1
2	1	2	2	2
3	1	3	3	3
4	2	1	2	3
5	2	2	3	1
6	2	3	1	2
7	3	1	3	2
8	3	2	1	3
9	3	3	2	1

A to D represent different hyperparameters, and the numbers 1, 2, and 3 within the table indicate levels of hyperparameter settings: levels 1, 2, and 3, respectively.

**Table 2:** Two-factor experimental design.

Independent Variable		Dependent Variable
A	B	Y
1	1	A_1_B_1_
1	2	A_1_B_2_
1	3	A_1_B_3_
2	1	A_2_B_1_
2	2	A_2_B_2_
2	3	A_2_B_3_

**Table 3:** Feature description of the Kaggle Cardiovascular Disease dataset.

No	Feature	Data Type
1	Age	int (days)
2	Height	int (cm)
3	Weight	float (kg)
4	Gender	categorical code
5	Systolic blood pressure	int
6	Diastolic blood pressure	int
7	Cholesterol	1: normal, 2: above normal, 3: well above normal
8	Glucose	1: normal, 2: above normal, 3: well above normal
9	Smoking	binary
10	Alcohol intake	binary
11	Physical activity	binary
12	Presence (or absence) of cardiovascular disease	binary

**Table 4:** Model hyperparameters and their levels in the *L*_18_(2^1^ × 3^7^) orthogonal array.

Hyperparameter	Hidden Layers	Activation Function	Optimizer	Learning Rate	Moment Rate	Hidden Nodes
	X_1_	X_2_	X_3_	X_4_	X_5_	X_6_
Level 1	4	logistic	lbfgs	0.2	0.7	4
Level 2	8	tanh	sgd	0.3	0.8	8
Level 3	12	relu	adam	0.4	0.9	12

**Table 5:** Taguchi method *L*_18_(2^1^ × 3^7^) and results.

Exp.	Hyperparameter						Accuracy			Average Accuracy	Standard Deviation	SN Ratio
	X_1_	X_2_	X_3_	X_4_	X_5_	X_6_	N_1_	N_2_	N_3_			
1	4	logistic	lbfgs	0.20	0.70	4	0.4951	0.4951	0.4951	0.4951	0.0000	−6.105
2	4	tanh	sgd	0.30	0.80	8	0.7389	0.7382	0.7262	0.7345	0.0071	−2.682
3	4	relu	adam	0.40	0.90	12	0.4951	0.5049	0.4951	0.4984	0.0056	−6.050
4	8	logistic	lbfgs	0.30	0.80	12	0.4951	0.4951	0.4951	0.4951	0.0000	−6.105
5	8	tanh	sgd	0.40	0.90	4	0.7404	0.7405	0.7331	0.7380	0.0043	−2.639
6	8	relu	adam	0.20	0.70	8	0.4951	0.4951	0.4951	0.4951	0.0000	−6.105
7	12	logistic	sgd	0.20	0.90	8	0.4951	0.5049	0.5049	0.5016	0.0056	−5.994
8	12	tanh	adam	0.30	0.70	12	0.4951	0.4951	0.5136	0.5013	0.0106	−6.002
9	12	relu	lbfgs	0.40	0.80	4	0.7341	0.7414	0.4951	0.6569	0.1401	−4.124
10	4	logistic	adam	0.40	0.80	8	0.4951	0.4951	0.4951	0.4951	0.0000	−6.105
11	4	tanh	lbfgs	0.20	0.90	12	0.7361	0.7386	0.7372	0.7373	0.0013	−2.647
12	4	relu	sgd	0.30	0.70	4	0.7389	0.7381	0.7404	0.7391	0.0012	−2.626
13	8	logistic	sgd	0.40	0.70	12	0.5049	0.4951	0.4951	0.4984	0.0056	−6.050
14	8	tanh	adam	0.20	0.80	4	0.4951	0.5049	0.4951	0.4984	0.0056	−6.050
15	8	relu	lbfgs	0.30	0.90	8	0.7416	0.7412	0.7404	0.7411	0.0007	−2.603
16	12	logistic	adam	0.30	0.90	4	0.4951	0.4951	0.4951	0.4951	0.0000	−6.105
17	12	tanh	lbfgs	0.40	0.70	8	0.7441	0.7418	0.7423	0.7427	0.0012	−2.584
18	12	relu	sgd	0.20	0.80	12	0.7384	0.7240	0.7431	0.7352	0.0100	−2.674
										0.5999	0.0110	−4.625

Exp., experiment; SN, signal-to-noise.

**Table 6:** Hyperparameter response table of average accuracies for *L*_18_(2^1^ × 3^7^).

Level	X_1_	X_2_	X_3_	X_4_	X_5_	X_6_
1	0.6166	0.4968	0.6447	0.5771	0.5786	0.6038
2	0.5777	0.6587	0.6578	0.6177	0.6025	0.6184
3	0.6055	0.6443	0.4972	0.6049	0.6186	0.5776
Effect	0.0389	0.1619	0.1605	0.0406	0.0400	0.0408
Rank	6	1	2	4	5	3

The effect of a factor is defined as the maximum difference in the overall average accuracy across various levels. The hyperparameter response table shows the analysis results for all the hyperparameter effects.

**Table 7:** Hyperparameter ANOVA table of average accuracies for *L*_18_(2^1^ × 3^7^).

Source	DF	SS	MS	*F*	*p*
X_1_	2 *	0.004817 *			
X_2_	2	0.096382	0.048191	12.35	0.001
X_3_	2	0.095378	0.047689	12.23	0.001
X_4_	2 *	0.005165 *			
X_5_	2 *	0.00485 *			
X_6_	2 *	0.005116 *			
Error	(13)	(0.050712)	0.003901		
Sum	17	0.242471			
				*R* ^2^	*R*^2^ (adj.)
				79.09%	72.65%

ANOVA, analysis of variance; DF, degrees of freedom; SS, sum of squares; MS, mean of squares; adj., adjusted. The asterisk (*) signifies pooling values in the error term.

**Table 8:** Hyperparameter response table of the SN ratio for *L*_18_(2^1^ × 3^7^).

Level	X_1_	X_2_	X_3_	X_4_	X_5_	X_6_
1	−4.3692	−6.0775	−4.0281	−4.9292	−4.9120	−4.6083
2	−4.9254	−3.7673	−3.7773	−4.3538	−4.6234	−4.3454
3	−4.5805	−4.0303	−6.0697	−4.5920	−4.3397	−4.9214
Effect	0.5562	2.3102	2.2924	0.5754	0.5723	0.5760
Rank	6	1	2	4	5	3

SN, signal-to-noise. The effect of a factor is defined as the maximum difference in the overall average accuracy across various levels. The hyperparameter response table shows the analysis results for all the hyperparameter effects.

**Table 9:** Hyperparameter ANOVA table of the SN ratio for *L*_18_(2^1^ × 3^7^).

Source	DF	SS	MS	*F*	*p*
X_1_	2 *	0.946 *			
X_2_	2	19.1948	9.5974	11.78	0.001
X_3_	2	18.9717	9.4859	11.64	0.001
X_4_	2 *	1.0031 *			
X_5_	2 *	0.9827 *			
X_6_	2 *	0.9979 *			
Error	(13)	(10.5947)	0.815		
Sum	17	48.7612			
				*R* ^2^	*R*^2^ (adj.)
				78.27%	71.59%

ANOVA, analysis of variance; SN, signal-to-noise; DF, degrees of freedom; SS, sum of squares; MS, mean of squares; adj., adjusted. The asterisk (*) signifies pooling values in the error term.

**Table 10:** Results of confirmation experiment for *L*_18_(2^1^ × 3^7^).

Method	N_1_	N_2_	N_3_	N_4_	N_5_	Average Accuracy	Standard Deviation	SN Ratio
*L*_18_(2^1^ × 3^7^)	0.7398	0.7392	0.7394	0.7369	0.7361	0.7383	0.0017	−2.636

SN, signal-to-noise.

**Table 11:** Model hyperparameters and their levels in the *L*_9_(3^4^) orthogonal array.

Hyperparameter	Learning Rate	Moment Rate	Hidden Nodes
	X_4_	X_5_	X_6_
Level 1	0.25	0.85	6
Level 2	0.3	0.9	8
Level 3	0.35	0.95	10

**Table 12:** Taguchi method *L*_9_(3^4^) and results.

Exp.	Hyperparameter			Accuracy			Average Accuracy	Standard Deviation	SN Ratio
	X_4_	X_5_	X_6_	N_1_	N_2_	N_3_			
1	0.25	0.85	6	0.7390	0.7379	0.7417	0.7395	0.0020	−2.621
2	0.25	0.90	8	0.7392	0.7381	0.7376	0.7383	0.0008	−2.635
3	0.25	0.95	10	0.7348	0.7414	0.7398	0.7387	0.0035	−2.631
4	0.30	0.85	8	0.7348	0.7411	0.7399	0.7386	0.0033	−2.632
5	0.30	0.90	10	0.7399	0.7341	0.7400	0.7380	0.0034	−2.639
6	0.30	0.95	6	0.7357	0.7360	0.7395	0.7371	0.0021	−2.650
7	0.35	0.85	10	0.7418	0.7349	0.7388	0.7385	0.0035	−2.633
8	0.35	0.90	6	0.7376	0.7301	0.7374	0.7350	0.0043	−2.675
9	0.35	0.95	8	0.7420	0.7374	0.7291	0.7361	0.0065	−2.661
							0.7378	0.0033	−2.642

Exp., experiment; SN, signal-to-noise.

**Table 13:** Hyperparameter response table of average accuracies for *L*_9_(3^4^).

Level	X_4_	X_5_	X_6_
1	0.7388	0.7389	0.7372
2	0.7379	0.7371	0.7377
3	0.7365	0.7373	0.7384
Effect	0.0023	0.0017	0.0012
Rank	1	2	3

The effect of a factor is defined as the maximum difference in the overall average accuracy across various levels. The hyperparameter response table shows the analysis results for all the hyperparameter effects.

**Table 14:** Hyperparameter ANOVA table of average accuracies for *L*_9_(3^4^).

Source	DF	SS	MS	*F*	*p*-Value
X_4_	2	0.000008	0.000004	5.94	0.06
X_5_	2	0.000006	0.000003	4.12	0.10
X_6_	2 *	0.000002 *			
Error	(4)	(0.000003)	0.000001		
Sum	8	0.000016			
				*R* ^2^	*R*^2^ (adj.)
				83.41%	66.83%

ANOVA, analysis of variance; DF, degrees of freedom; SS, sum of squares; MS, mean of squares; adj., adjusted. The asterisk (*) signifies pooling values to the error term.

**Table 15:** Hyperparameter response table of the SN ratio for *L*_9_(3^4^).

Level	X_4_	X_5_	X_6_
1	−2.629	−2.629	−2.648
2	−2.640	−2.650	−2.643
3	−2.656	−2.648	−2.634
Effect	0.027	0.021	0.014
Rank	1	2	3

SN, signal-to-noise. The effect of a factor is defined as the maximum difference in the overall average accuracy across various levels. The hyperparameter response table shows the analysis results for all the hyperparameter effects.

**Table 16:** Hyperparameter ANOVA table of the SN ratio for *L*_9_(3^4^).

Source	DF	SS	MS	*F*	*p*-Value
X_4_	2	0.001133	0.000566	6.07	0.06
X_5_	2	0.000773	0.000386	4.14	0.10
X_6_	2 *	0.000299 *			
Error	(4)	(0.000373)	0.000093		
Sum	8	0.002279			
				*R* ^2^	*R*^2^ (adj.)
				83.62%	67.23%

ANOVA, analysis of variance; SN, signal-to-noise; DF, degrees of freedom; SS, sum of squares; MS, mean of squares; adj., adjusted. The asterisk (*) signifies pooling values into the error term.

**Table 17:** Results of confirmation experiment for *L*_9_(3^4^).

Method	N_1_	N_2_	N_3_	N_4_	N_5_	Average Accuracy	Standard Deviation	SN Ratio
*L*_9_(3^4^)	0.7401	0.7410	0.7439	0.7415	0.7407	0.7414	0.0015	−2.598

SN, signal-to-noise.

**Table 18:** Comparison table between *L*_18_(2^1^ × 3^7^) and *L*_9_(3^4^).

Comparison	Hidden Layers	Activation Function	Optimizer	Learning Rate	Moment Rate	Hidden Nodes	Average Accuracy	Standard Deviation	SN Ratio
	X_1_	X_2_	X_3_	X_4_	X_5_	X_6_			
*L*_18_(2^1^ × 3^7^)	4	tanh	sgd	0.3	0.9	8	0.7383	0.0017	−2.636
*L*_9_(3^4^)	4	tanh	sgd	0.25	0.85	10	0.7414	0.0015	−2.598
						Improvement	0.0032	−0.0002	0.0374

SN, signal-to-noise.

**Table 19:** Comparison between the TSTO and other state-of-the-art methods [11].

Method	Accuracy
ANN	0.6835
Logistic regression	0.7235
Decision tree	0.6172
Random forest	0.6894
Support vector machine	0.7216
K-Nearest Neighbor	0.6834
GA-ANN	0.7343
First-stage Taguchi method	0.7383
Proposed TSTO	0.7414

TSTO, two-stage Taguchi optimization; ANN, artificial neural network; GA, genetic algorithm.

## Data Availability

The data used in the study are publicly available from Kaggle. Available: https://www.kaggle.com/datasets/sulianova/cardiovascular-disease-dataset (accessed on 17 February 2023).
